# Drug treatment of heart failure in the elderly

**DOI:** 10.1007/s00059-017-4668-9

**Published:** 2018-01-16

**Authors:** D. Berliner, J. Bauersachs

**Affiliations:** Dept. of Cardiology and Angiology, Hannover Medical School, Carl-Neuberg-Str. 1, 30625 Hannover, Germany

**Keywords:** Heart failure, Treatment, Aged, Comorbidity, Drug interactions, Herzinsuffizienz, Therapie, Alte Patienten, Komorbiditäten, Arzneimittelinteraktionen

## Abstract

The prevalence of heart failure increases with age. Changes in the age distribution and growing life expectancy will lead to a further rise. However, data concerning drug treatment of heart failure especially in the elderly are scarce. Subgroup analyses of the heart failure trials suggest that drug therapy in older patients should follow the recommendations in the current guidelines. In doing so, several common comorbidities in these patients (e. g., impaired renal function) have to be considered and may have an influence on the therapy (e. g., drug dose, choice of active pharmaceutical ingredient, etc.). Especially in old, multimorbid patients, possible interaction of drugs might play a substantial role. In many cases the main goal of the therapy, especially in the very elderly, is to improve symptoms and quality of life.

## Zusammenfassung

Die Prävalenz der Herzinsuffizienz nimmt mit steigendem Alter deutlich zu. Aufgrund von Veränderungen in der Altersstruktur und einer zunehmenden Lebenserwartung ist mit einer weiteren Zunahme in den kommenden Jahren zu rechnen. Daten zur medikamentösen Therapie der Herzinsuffizienz speziell bei älteren Patienten existieren nur zu einem geringen Maße. Subgruppenanalysen aus den großen Herzinsuffizienzstudien legen jedoch nahe, dass die Therapie sich an der aktuellen leitliniengerechten Therapie der Herzinsuffizienz orientieren sollte. Dabei müssen jedoch Komorbiditäten wie v. a. Einschränkungen der Nierenfunktion und der mentale Status des Patienten mit ins Kalkül genommen werden und ggf. zur Anpassung der Therapie (z. B. Dosierung, Wirkstoffauswahl etc.) führen. Gerade bei älteren, multimorbiden Patienten spielen Arzneimittelinteraktionen eine wesentliche Rolle. Ziel der Therapie ist in vielen Fällen v. a. die Verbesserung der Symptomatik sowie der Lebensqualität.

## Increasing prevalence of heart failure in the elderly

**Fig. 1:**
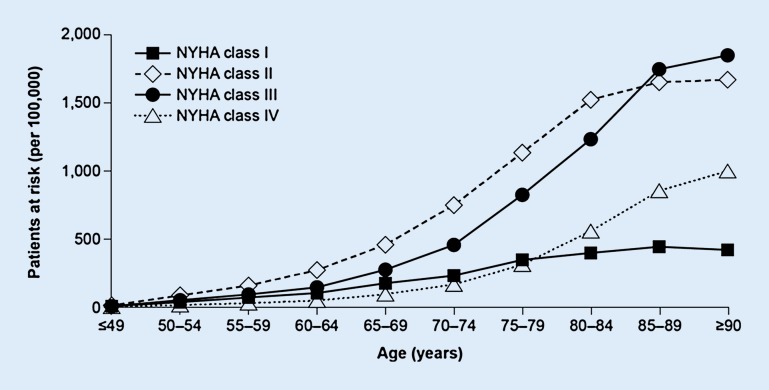
Prevalence of heart failure diagnoses by age group according to the NYHA classification (from [41], licensed under the terms of the Creative Commons Attribution 4.0 International License: http://creativecommons.org/licenses/by/4.0/). *NYHA* New York Heart Association

The syndrome of chronic heart failure (HF) is a growing problem due to better medical care and increasing life expectancy (Fig. 1). Exact numbers regarding the prevalence of HF in Germany are limited because of the inconsistent definitions of HF. However, the prevalence of HF is highly dependent on age. Thus, in the age group of 45–55 years prevalence is below 1%, whereas prevalence increases to approximately 10% in patients aged over 80 years [1]. In patients aged >65 years with dyspnea on exertion presenting to their general practitioner, a sixth will have unrecognized HF [2]. Besides, mortality in elderly patients with HF is severely increased: Data from the United States show that the mean survival time in older patients with HF is 2.5 years, with 25% dying in the first 12 months [3].

Furthermore, differences exist regarding the type of HF and the relation of gender. In younger age, most patients suffer from systolic HF (HFrEF: HF with reduced ejection fraction [2]), and men are affected more often than women. In older patients, women are affected more frequently. The percentage of diastolic HF (HFpEF: HF with preserved ejection fraction) is higher in the elderly and the ratio of genders is balanced [4].

HF is mostly caused by coronary artery disease and hypertension. Moreover, in older patients, other pathophysiologic factors contribute to development of HF [3]:

- Dilatation of the left ventricle
- Reduced/limited diastolic function
- Diminished elasticity of the aorta, altered cardiovascular coupling
- Increased dependency of the diastolic filling from the atrial contraction
- Increased variability of the cardiac output according to volume status

## Altered clinical presentation of HF in the elderly

Typical signs and symptoms of HF comprise of dyspnea, fatigue, ankle swelling, and edema [2, 5]. The difficulty of diagnosing HF only on the basis of clinical criteria was shown in a prospective and randomized trial with 305 patients. The investigators were able to diagnose or rule out HF based on clinical presentation, medical history, and examination only in 52% [6]. In elderly patients this challenge is even more demanding as patients frequently present with atypical, nonspecific symptoms such as tiredness, altered mental status, depression, and loss of appetite [3, 5]. In a study by Oudejans et al., in only 50% of geriatric patients with suspected HF could the diagnosis be confirmed, and typical signs of HF were absent in one third of patients with HF [5].

In the current HF guidelines of the European Society of Cardiology (ESC) the natriuretic peptides B‑type natriuretic peptide (BNP) and the N‑terminal end of the propeptide (NT-proBNP) play a pivotal role in diagnosing HF [2]. Natriuretic peptides are released from the ventricular myocardium as a consequence of increased wall stress [7]. In this context it has to be recognized that levels of natriuretic peptides increase with age [8]. Established reference values for the elderly do not exist. Furthermore, it has to be acknowledged that comorbidities like atrial fibrillation and chronic renal insufficiency have a significant influence on natriuretic peptide levels. Nevertheless, owing to a sensitivity of approximately 90%, natriuretic peptides are useful in ruling out HF [8]. Yet, the gold standard in diagnosing HF is echocardiography.

## Drug treatment of HF with reduced ejection fraction

In most trials investigating drug treatment of HF, older patients are not adequately represented. Therefore, recommendations for the treatment of this cohort are more or less based on subgroup analysis and expert opinions. In general, pharmacological treatment of HF patients is mainly based on beta-blockers and angiotensin-converting enzyme (ACE) inhibitors (ACEi) apart from diuretics.

### Diuretics

Diuretic therapy is the basis of drug therapy in symptomatic HF. It clearly improves symptoms and quality of life [9]. Diuretics are used in an acute setting for patients with volume overload in usually higher doses for the amelioration of symptoms (e. g., dyspnea, edema) and in patients with compensated HF to maintain a stable state (i. e., “weight”). The dose of diuretics should be as low as necessary, at the minimum effective dose, to reach and keep euvolemia. In the course of the disease, the potential for dose reductions should be checked regularly [2]. Especially in the elderly, confusion is frequently a consequence of fluid depletion due to restriction and the additional use of diuretics. Furthermore, it may be caused by hyponatremia as a consequence of the diuretic therapy [4].

### Beta-blockers

Two randomized trials have investigated the value of beta-blockers in elderly patients with HF. In the SENIORS trial, therapy with nebivolol was compared with placebo. Mean age in this study was 76 years. Therapy with nebivolol led to a significant reduction of the primary endpoint all-cause mortality and cardiovascular hospitalizations (31.1% vs. 35.3%; relative risk reduction 12% [10]). The CIBIS-ELD trial compared therapy with the beta-blockers bisoprolol and carvedilol in older patients (mean age 73 years). No differences were found regarding tolerance or achieved target dose, but patients with bisoprolol more often suffered from bradycardias whereas carvedilol led to a reduction in the forced expiratory volume (FEV_1_) [11]. This should be taken into account when choosing the “individual” beta-blocker. Furthermore, a later analysis of the CIBIS-ELD trial revealed that heart rate after up-titration, but not the dose of the beta-blocker, predicted all-cause mortality risk [12]. Elderly patients with a heart rate in the range of 55–64 bpm had the lowest mortality [12]. In the MERIT-HF trial, therapy with metoprolol succinate was compared with placebo in patients with HF. The study enrolled patients up to an age of 80 years and included a considerable percentage of elderly patients. A retrospective subgroup analysis found a similar reduction regarding mortality and morbidity in patients 69 years or older compared with those younger than 69 years [3, 13].

### ACE inhibitors/angiotensin receptor blocker

Randomized controlled studies in elderly patients with ACEi or angiotensin receptor blocker (ARB) do not exist. In the CONSENSUS trial (enalapril vs. placebo), mortality was significantly reduced in the enalapril arm (26% vs. 44% after 6 months). The mean age in this trial was 71 years, which means that a considerable percentage of elderly patients were enrolled [14]. Thus, a benefit for older patients can be deduced from this trial. Observational studies and a meta-analysis of studies in patients after myocardial infarction with HF confirm these findings [3].

To avoid severe hypotension or renal insufficiency, ACEi should be started in low doses after correction of hyponatremia or volume depletion in the elderly [15]. The dose of the diuretics might have to be raised transiently after reaching the maintenance dose of the ACEi [15]. In the further course of treatment, diuretics might be reduced again.

### Mineralocorticoid receptor antagonists (formerly aldosterone antagonists)

Since the RALES trial [16], the EPHESUS trial [17], and the EMPHASIS-HF trial [18], therapy with mineralocorticoid receptor antagonists (MRA) for patients with symptomatic HFrEF despite therapy with an ACEi and a beta-blocker is established and implemented in the guidelines. Randomized controlled trials with MRA in the elderly with HF also have not been performed. However, prespecified subgroup analyses both in the RALES and in the EMPHASIS-HF trial have shown that older HF patients benefit from treatment with an MRA to a similar extent as younger patients [16, 18, 19].

The most important adverse effect of MRA treatment is hyperkalemia. Particularly in older patients, renal markers and electrolytes should be checked regularly—especially with concomitant medication with an ACEi or an ARB. Higher age is an independent risk factor for developing hyperkalemia [3].

In the near future potassium binders like patiromer might help in reaching adequate HF medication despite the tendency toward hyperkalemia. Patiromer is a polymer that acts as an ion exchanger in the colon. The PEARL-HF trial enrolled 105 HF patients with a history of hyperkalemia resulting in discontinuation of the HF medication. In the patiromer group, potassium was significantly lowered resulting in higher dosages of the HF medication (i. e., spironolactone dose) [20].

### I_f_-channel inhibitor ivabradine

Through inhibition of the I_f_ channel of the sino-atrial node, ivabradine slows the heart rate in sinus rhythm. In the SHIFT trial, additional administration of ivabradine on top of optimized HF medication (incl. beta-blocker) led to a significant decrease in HF hospitalizations and cardiovascular mortality (primary endpoint, relative risk reduction: 18%) [21] resulting in a corresponding recommendation in the current guidelines [2]. Likewise, for ivabradine no randomized study exists concerning efficacy in the elderly. However, in a subgroup analysis the efficacy and safety of ivabradine were evaluated across the age spectrum: Patients were divided into four groups (<53, 53–60, 60–69, and >69 years), and ivabradine use was associated with a relative risk reduction of the primary endpoint with no statistical difference in the elderly [22]. The authors conclude that, “age does not limit the appropriate use of ivabradine in patients with chronic HF and systolic dysfunction” [22].

### Angiotensin receptor-neprilysin inhibitor

In the past few years, a new drug class of “angiotensin receptor-neprilysin inhibition (ARNI)” emerged in HF therapy. The first and to date only substance in this class is “LCZ696” and comprises an angiotensin receptor blocker (ARB, valsartan) and sacubitril, which is an inhibitor of the neutral endopeptidase (neprilysin) reducing degradation of natriuretic peptides. The PARADIGM-HF trial compared therapy with sacubitril/valsartan with therapy with the ACEi enalapril [23]. The primary endpoint consisted of cardiovascular mortality and HF hospitalizations and was highly significantly reduced in the sacubitril/valsartan group (−20%). Furthermore, a significant reduction was shown for cardiovascular mortality (−20%), all-cause mortality (−16%), and HF hospitalizations (−21%). The overwhelming effects have resulted in the recommendation for an ARNI in the current guidelines for all patients who remain symptomatic despite therapy with an ACEi (or ARB), a beta-blocker, and an MRA [2, 24]. Regarding the elderly, the authors of a recent subgroup analysis stated that LCZ696 was more beneficial than enalapril across the spectrum of age in the PARADIGM-HF trial, with a favorable benefit–risk profile in all age groups including the elderly [25]. Besides, typical side effects of the therapy (hypotension, renal impairment, hyperkalemia) were similar in the age categories analyzed [25]. It should be kept in mind, especially regarding older patients, that sacubitril/valsartan provokes a significantly higher incidence of symptomatic hypotension than does therapy with an ACEi. Thus, patients with very low blood pressure during ACEi treatment should not be switched to an ARNI [26].

### Digitalis

Maison et al. reported that digitalis is prescribed more frequently in older HF patients (>75 years) than in younger patients (≤75 years) at hospital discharge [27]. Overall the role and significance of cardiac glycosides in the treatment of chronic HF is currently still unclear [28]. There is one prospective, randomized study with digoxin (DIG trial) in patients with HFrEF [29], which was conducted before the current HF medication was established (i. e., very low rate of concomitant therapy with beta-blocker and MRA). Hospitalizations for HF were significantly reduced in the digoxin group whereas total mortality was not influenced. A subgroup analysis of the DIG trial showed that in patients with lower serum levels of digoxin (0.5–0.9 ng/ml), total mortality was significantly reduced in contrast to patients with high levels (excess mortality) [30]. Especially patients with advanced HF (NYHA III–IV, LVEF <25%) and patients with atrial fibrillation and high ventricular rate seem to benefit from the therapeutic use of cardiac glycosides regarding mortality and hospitalization rates [31]. A subgroup analysis of the DIG trial showed that digoxin reduced the 30-day all-cause hospital admission in older patients with chronic HF and indicated a trend to a lower 30-day all-cause mortality in those patients [32]. Owing to the narrow therapeutic range of cardiac glycosides, they should be used with caution especially in women and older patients, and digitoxin should be preferred particularly in patients with impaired renal function [2].

There are no trials to date on digitoxin or the effect of digitalis in HF patients with atrial fibrillation. A large randomized study investigating the role of digitoxin in patients with HF on contemporary drug therapy is under progress: the DIGIT-HF trial (*Dig*italis to *I*mprove Ou*t*comes in Patients with Advanced Systolic Chronic *H*eart *F*ailure, EudraCT-No.: 2013-005326-38).

### Treatment approach

Generally, international guidelines consistently recommend that the drug therapy for elderly HF patients should be based on beta-blockers and ACEi (or ARB) [2–4]. Furthermore, addition of an MRA should be considered. Cardiac glycosides may improve symptoms in those patients but should be used with caution especially in patients with reduced renal function to prevent intoxications. Digitoxin should be used rather than digoxin in such patients [4]. Individual doses of diuretics, normally loop diuretics, should be used to keep volume homeostasis. Electrolytes and renal function should be controlled on a regular basis.

**Fig. 2:**
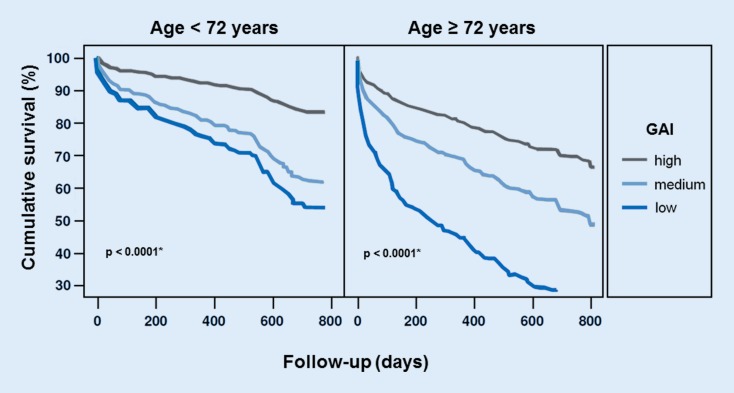
Impact of guideline-conforming drug therapy on all-cause mortality depending on patients’ age (heart failure with reduced ejection fraction, *n* = 637); Interdisciplinary Network Heart Failure prospective cohort study (Würzburg, Germany). Graphs for all-cause death plotted from Cox proportional hazards regression. *Adjustment was made for sex, NYHA functional class, C‑reactive protein, anemia, renal dysfunction, and body mass index. *GAI* guideline adherence indicator (range 0–100%). The GAI considers intake of life-saving substance classes (i. e., betablocker, angiotensin-converting enzyme inhibitor/angiotensin receptor blocker, mineralocorticoid receptor antagonists) including respective contraindications per substance class (data based on original data published in [33]; courtesy of Prof. Dr. S. Störk, printed with permission)

Data from the INH registry (interdisciplinary network for heart failure) showed clearly that older patients in particular benefit from a pharmacological therapy according to the guidelines ([33]; Fig. 2).

## Treatment of patients with HF with preserved ejection fraction

A considerable percentage of elderly patients with HF have HFpEF [34]. To date, no randomized trial could show a clear benefit of any drug therapy regarding mortality in patients with HFpEF irrespective of the patients’ age [2]. According to the statements in the guidelines, the main therapeutic goal in patients with HFpEF is improvement of symptoms (edema, dyspnea) and subjective well-being. The same is true for the elderly. An adequately dosed therapy with diuretics is recommended to reach this target. In patients with sinus rhythm, treatment with nebivolol, spironolactone, or candesartan was able to reduce HF hospitalizations [2]. Besides, it is important to note that the causes of hospitalization and mortality in HFpEF patients are frequently noncardiovascular. Screening for comorbidities and their adequate treatment are a major recommendation of the current guidelines.

## Comorbidities and polypharmacy

Comorbidities are common in HF patients and have received more attention during the past few years [2]. Especially in the elderly, comorbidities play an important role also for prognosis. In the INH registry, approximately 50% of the patients had seven or more comorbidities [4] and a significant association was found between the number of comorbidities and the risk for all-cause mortality in those patients [35]. The higher number of comorbidities impedes drug therapy of HF and augments the complexity of the condition. Polypharmacy is common, which increases the hazard of drug interactions and drug-related adverse effects [36]. Also, phytotherapeutics and dietary supplements may interact with evidence-based HF drugs and lower their effectiveness. Dietary supplements without proven efficacy, such as Crataegus, coenzyme Q10, *Terminalia arjuna*, carnitine, or taurine should not be administered additionally [4]. Another point that has to be acknowledged is that adherence to drug therapy decreases with the number of drugs prescribed. This problem is exacerbated in patients with dementia [4]. Furthermore, some drugs typically used in common comorbidities are known to negatively impact the prognosis of HF patients [2]. Particularly drugs that aggravate the symptoms by impairing myocardial contractility or causing fluid retention should not be used. Typical drugs that should be avoided in patients with HF are [2, 4]:

- Nonsteroidal anti-inflammatory drugs and cyclo-oxygenase-2 inhibitors (sodium and water retention, worsening of kidney function, worsening of HF, increase in hospitalizations).
- Glitazones (worsening of HF).
- Calcium channel blocker, excluding amlodipine and felodipine (negative inotropic effect, worsening of HF, increase in hospitalizations).
- Dronedarone for rhythm control in AF (increased risk of cardiovascular events, increased mortality).
- Class I antiarrhythmic agents (increased mortality).
- Moxonidine (increased mortality).
- Tricyclic antidepressants (worsening of HF, arrhythmias, second- and third-degree heart block, sick sinus syndrome).
- Alpha-blockers (neuro-humoral activation, water retention, worsening of HF) should not be used in the treatment of benign prostate hyperplasia and in the treatment of hypertension only after exploiting other treatment strategies.
- Corticosteroids (sodium and water retention) should be administered in the lowest justifiable dose under suitable surveillance.

Furthermore, the following drug combinations should be avoided [2]:

- Combination of ivabradine, ranolazine, and nicorandil (unclear safety)
- Combination of nicorandil and nitrates (missing additional effect)
- Adding an ARB to an ACEi and an MRA (increased risk of hyperkalemia, possible worsening of kidney function)

## General considerations

**Fig. 3:**
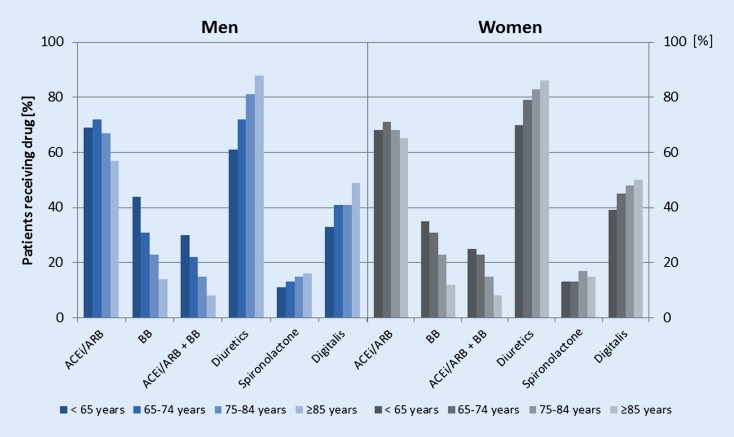
Prescription of cardiovascular drugs in patients with heart failure according to sex and age (modified from [39]). *ACEi* angiotensin-converting enzyme inhibitor, *ARB* angiotensin receptor blocker, *BB* beta-blocker

The aforementioned study of Oudejans et al. [5] showing misdiagnosed HF in approximately 50% of elderly patients underlines the need for performing echocardiography on all patients with suspected HF to confirm the diagnosis. In contrast to younger patients, elderly patients with HF more often are treated by general practitioners than by cardiologists. Typically, these patients are frequently female and have HFpEF. It has been shown that general practitioners use fewer additional investigations and prescribe less potentially beneficial medication than do cardiologists [37]. In the Euro Heart Failure Survey II, underuse and underdosage of medications recommended for HF were described in octogenarians with HFrEF (prescription rates of 82% for ACEi/ARB, 56% for beta-blocker, and 54% for MRA) [38]. But the authors found significant improvement in contrast to prior surveys (e. g., Euro Heart Failure Survey I). These data have been confirmed by other studies showing that drugs such as beta-blockers and ACEi are less prescribed in eligible patients over 75 years of age ([27, 39]; Fig. 3). During hospital stay and during the first year after discharge, mortality rates were significantly increased in octogenarians compared with patients aged <80 years (10.7% vs. 5.6% and 28.4% vs. 18.5% respectively, *p* < 0.001) [38].

Different guidelines recommend that in patients suffering from multimorbidity, at least an ACEi and a beta-blocker should be prescribed whereas prescription of MRA and digitalis should be decided individually [4, 40]. Lower initial doses and slower dose increases may improve tolerance and result in better drug adherence [40]. Confusion is frequently a consequence of fluid depletion due to fluid restriction and use of diuretics or hyponatremia [4]. Patients presenting with newly diagnosed confusion should be screened for such conditions. Furthermore, patients with regular cardiac decompensations despite optimal drug therapy should be screened for signs of cognitive impairment or dementia [40]. Another problem in multimorbid patients is that contradictory advice by different medical specialists may result in confusion, nonadherence, and adverse outcomes [40].

The main goal of therapy especially in the very elderly is to improve symptoms and quality of life. Patients and their relatives should be involved in defining individual therapeutic goals.

## Conclusion

The prevalence of HF—especially HFpEF—increases in the elderly. Signs of HF and symptoms may differ from younger patients. Data on drug treatment for these patients are scarce but retrospective analyses suggest that older patients might benefit from the same recommendations as younger HF patients. In this context, typical comorbidities (e.g., renal insufficiency) must be taken into account. Further comorbidities such as cognitive impairment, dementia, and depression have a negative impact on therapy adherence and prognosis. As the number of elderly patients is steadily growing, further studies are necessary to elucidate the significance of a modern guideline-directed therapy in the elderly.
